# A multi-omic investigation of male lower urinary tract symptoms: Potential role for JC virus

**DOI:** 10.1371/journal.pone.0246266

**Published:** 2021-02-25

**Authors:** Samuel Thomas, Christopher D. Dunn, Lewis J. Campbell, Douglas W. Strand, Chad M. Vezina, Dale E. Bjorling, Kristina L. Penniston, Lingjun Li, William A. Ricke, Tony L. Goldberg

**Affiliations:** 1 Molecular and Environmental Toxicology Center, University of Wisconsin-Madison, Madison, Wisconsin, United States of America; 2 Department of Pathobiological Sciences, University of Wisconsin-Madison, Madison, Wisconsin, United States of America; 3 Department of Urology, UT Southwestern Medical Center, Dallas, Texas, United States of America; 4 George M. O’Brien Center of Research Excellence, University of Wisconsin-Madison, Madison, Wisconsin, United States of America; 5 Department of Urology, University of Wisconsin School of Medicine and Public Health, Madison, Wisconsin, United States of America; 6 School of Pharmacy, University of Wisconsin-Madison, Madison, Wisconsin, United States of America; 7 UW-Madison Global Health Institute, University of Wisconsin-Madison, Madison, Wisconsin, United States of America; King’s College London, UNITED KINGDOM

## Abstract

Male lower urinary tract symptoms (LUTS) comprise a common syndrome of aging that negatively impacts quality of life. The etiology of LUTS is multifactorial, involving benign prostatic hyperplasia, smooth muscle and neurologic dysfunction, inflammation, sexually transmitted infections, fibrosis, and potentially dysbiosis, but this aspect remains poorly explored. We investigated whether the presence of infectious agents in urine might be associated with LUTS by combining next-generation DNA sequencing for virus discovery, microbiome analysis for characterization of bacterial communities, and mass spectrometry-based metabolomics. In urine from 29 LUTS cases and 9 controls from Wisconsin, we found a statistically significant association between a diagnosis of LUTS and the presence of JC virus (JCV), a common neurotropic human polyomavirus (*Polyomaviridae*, *Betapolyomavirus*) linked to severe neurologic disease in rare cases. This association (based on metagenomics) was not borne out when specific polymerase chain reaction (PCR) testing was applied to this set of samples, likely due to the greater sensitivity of PCR. Interestingly, urine metabolomics analysis identified dysregulation of metabolites associated with key LUTS processes. Microbiome analysis found no evidence of microbial community dysbiosis in LUTS cases, but JCV-positive samples contained more *Anaerococcus* species, which are involved in polymicrobial infections of the urinary tract. Neither age nor body mass index were significantly associated with the presence of urinary JCV—in the initial group or in an additional, regionally distinct group. These data provide preliminary support the hypothesis that viruses such as JCV may play a role in the development or progression of LUTS, together with other infectious agents and host metabolic responses.

## Introduction

Male lower urinary tract symptoms (LUTS) include frequency, urgency, and pain with urination [1]. These symptoms result in a substantial cost to society, both in detriment to quality of life for the roughly 37% of men who will develop them by age 70 [2], and the total economic burden of over $3.9 B in the US annually [3]. The etiology of LUTS is considered to be multifactorial, including benign prostatic hyperplasia (BPH), smooth muscle and neurologic dysfunction, oxidative stress and inflammation, and fibrosis [1]. However, the factors precipitating these pathologic changes remain obscure.

Infectious agents may play a role in LUTS, but research into this topic has been limited [4]. The normal human urinary tract was once assumed sterile, but recent reports indicate that a urinary microbiome does exist [5–9]. The microbiome in general exerts pleiotropic effects on host biological processes, including key LUTS processes like oxidative stress [10] and inflammation [11]. Dysbiosis of the urinary tract, in turn, has been associated with urologic disease including bladder cancer [12], chronic kidney disease [13], and female LUTS [14]. In male LUTS, for example, a previous study observed that detectable bladder bacteria were associated with LUTS symptom severity, but found no association with microbial community composition [15]. The microbiome of the gastrointestinal tract has been studied far more extensively than that of the urinary tract [16], and with revelatory consequence. For example, the human gastrointestinal microbiome responds to environmental factors such as xenobiotics, diet, and lifestyle, potentially leading to dysbiosis and increased incidence of metabolic syndrome and irritable bowel disease, among other conditions [11]. Efforts to rebalance the microbiome are gaining momentum, such as in the case of persistent bowel infection by *Clostridium difficile*, for which fecal microbiota transplantation is being considered for approval as an investigative new drug [17]. Such approaches could apply to diseases linked to dysbiosis of the urinary tract microbiome as well.

To our knowledge, the role of viruses (key components of any microbiome [18]) in LUTS has not been explored directly. The average healthy human harbors >10 persistent viral infections at any time [18], but the consequences of such infection are currently unclear. Such viruses may be capable of contributing to key LUTS processes. For example, viruses generate oxidative stress [19], leading to DNA damage, inflammasome assembly, and even carcinogenesis in some cases [20]. In addition to oxidative stress and inflammatory processes, viruses have roles in fibrosis [21] and neuropathy [22]. In another study, genes involved in anti-viral processes were increased in symptomatic BPH, suggesting possible association of viruses with LUTS [23].

**Fig 1:**
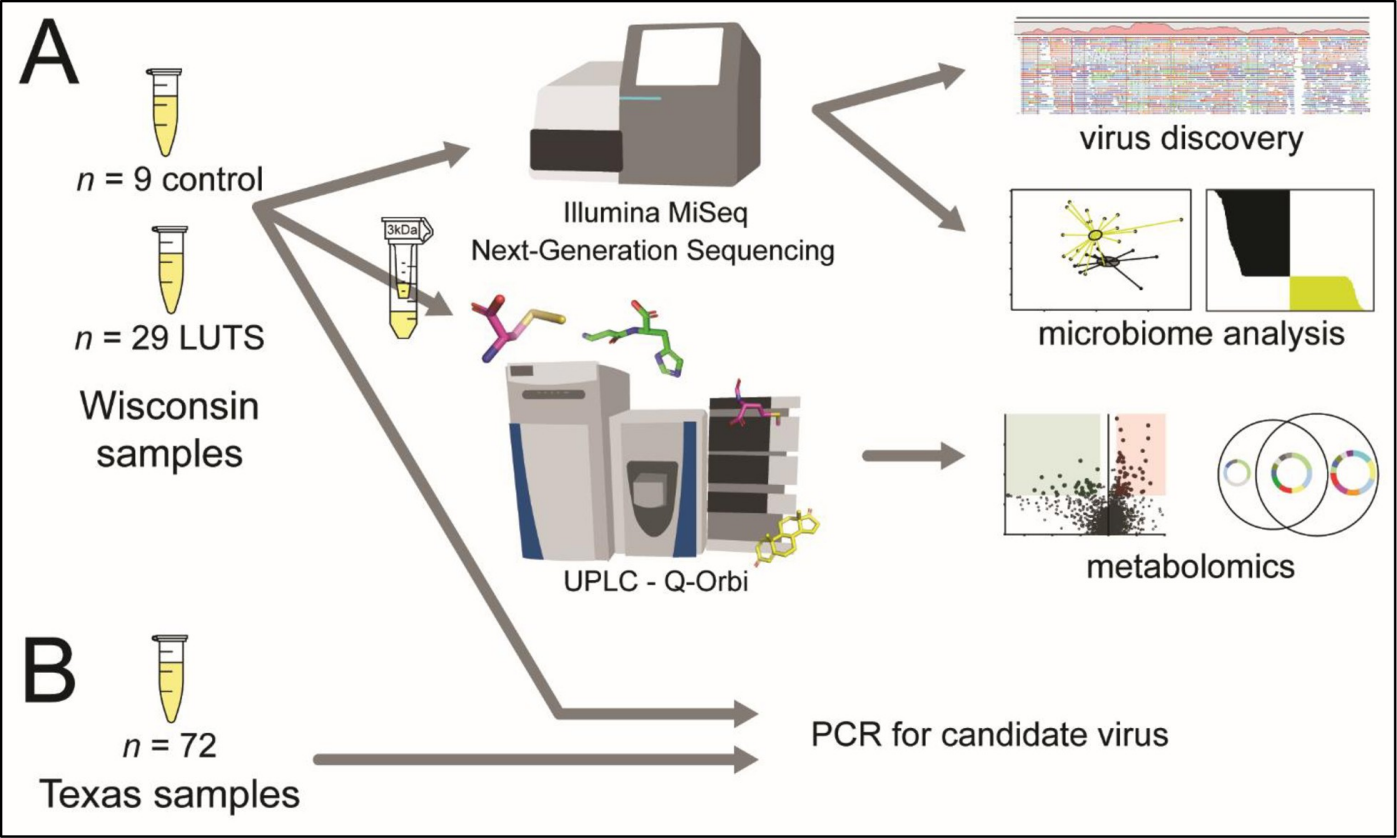
Experimental design and workflow. **A**: Next-generation sequencing to investigate viral associations with LUTS and microbiome and mass spectrometry-based metabolomics for molecular associations with virome in Wisconsin study. **B**: PCR for candidate virus in Wisconsin and Texas sample groups.

In the present study, we used new methods in metagenomics for virus discovery to identify and characterize viruses in the urine of LUTS patients and controls to test the hypothesis that viruses interact with the host and other microbes in ways that may precipitate LUTS, either directly or indirectly (Fig 1). We adopted a decidedly “multi-omic” approach, in that we combined our analyses of viruses with bacterial microbiomes to generate a comprehensive picture of the microbial community in urine. We then used metabolomics to characterize all detectable small molecules, or metabolites, in the same urine samples to examine whether metabolic pathways related to infection might be modulated in the case of infection with specific agents and the LUTS phenotype [24]. We and others have previously investigated the metabolome of LUTS, identifying features consistent with fibrosis in urine [25], and dysregulated amino acid metabolism in serum [26]. Furthermore, dysbiosis in other systems generates detectable metabolome-level perturbation, including increased glutathione with *Salmonella* infection in a mouse model [27] and increased glutathione and 1-methylnicotinamide with pesticide-induced dysbiosis in mice [28]. Metabolomic signatures of viral infection also exist, such as increases in the antioxidant glutathione [29] and dysregulated lipid and amino acid metabolites in Dengue virus infection [30]. We thus sought to identify tripartite relationships among viruses, bacteria, and metabolites, to better understand the etiology of LUTS and, more generally, to expand upon the notion of chronic urinary tract disease as a polymicrobial phenomenon.

## Materials and methods

### Donor recruitment and sample collection

#### Donor recruitment and characteristics

The Wisconsin study was conducted under the guidance of the University of Wisconsin-Madison Human Research Protection Program (HRPP) and was approved by the Institutional Review Board. Men with or without BPH/LUTS were included in the study. Exclusion criteria were: diabetes or chronic kidney disease, use of urinary catheter, use of urinary tract instrumentation within preceding 14 days, and use of antibiotic within preceding 2 months. In total, 38 Wisconsin men were recruited, 29 of whom showed symptoms of LUTS and 9 of whom were considered LUTS free, as determined via AUA symptom scores (Table 1). Significant differences were observed between LUTS and control samples with respect to age and AUA score, as expected, but not body mass index (BMI) (Table 1).

**Table 1:** Sample donor details of Wisconsin and Texas studies; significant differences are italicized (Student’s *t*-test, *α* = 0.05).

Sample group	LUTS status	Sample donor details			
		Sample size	Age (mean ± SD)	BMI (mean ± SD)	AUA score (mean ± SD)
Wisconsin	positive	29	*65*.*9 ± 11*.*2*	28.8 ± 4.7	*4*.*6 ± 2*.*2*
	negative	9	*56*.*3 ± 14*.*2*	27.4 ± 4.0	*2*.*8 ± 2*.*0*
Texas	*n/a*	72	55.4 ± 22.3	28.3 ± 5.0	*n/a*

The Texas study was conducted under the guidance of the UT Southwestern Medical Center Human Research Protection Program Office (HRPPO) and was approved by the Institutional Review Board. In total, samples from 72 Texas men were used to determine whether the prevalence of the candidate factor (JCV; see below) varied by region (Table 1). These samples were collected from men undergoing prostate reduction surgery for unmitigated LUTS and decedent men undergoing organ harvest at the Southwest Transplant Alliance.

#### Urine sample collection

In the Wisconsin study, urine samples were collected consistent with a “clean-catch” protocol (*i*.*e*., with instructions to wash hands and foreskin with alcohol swabs and to deposit mid-stream urine into a sterile container). In the Texas study, urine was collected from patients undergoing prostate reduction surgery at pre-surgical testing or via catheter during surgery. Urine from decedent men was collected via catheter. Samples were frozen at -80°C until further processing.

#### Urine sample preparation

Urine samples were centrifuged at 10,000 *g* for 10 min to pellet sediments. Aliquots of supernatants and pellets were stored at -80°C until analysis.

### Next-generation sequencing

#### Viromes

Virus discovery was performed on urine supernatants following previously published methods [31, 32]. Briefly, viral nucleic acids were isolated using the AllPrep PowerViral DNA/RNA Kit (Qiagen, Hilden, Germany), and RNA was converted to double stranded cDNA using random hexamers. Libraries were prepared for sequencing on an Illumina MiSeq intrument (V3 chemistry, 600 cycle kit; Illumina, San Diego, CA, USA), using the Nextera XT DNA sample preparation kit (Illumina, San Diego, CA, USA). Sequence data were analyzed using CLC Genomics Workbench version 11.0 (CLC bio, Aarhus, Denmark). Low-quality bases (phred score < 30) were trimmed, and short reads (< 75 bp) were discarded along with reads that mapped to the human genome. To identify viruses, individual reads and contiguous sequences (“contigs”) assembled from individual reads were queried against Genbank and custom virus databases as previously described [31, 32].

Based on results from metagenomic analyses (see below), specific PCR was conducted for JCV. A published semi-nested PCR was used for this purpose following previously described methods [33] using urine supernatants (1 mL each) from which nucleic acids were extracted with the AllPrep PowerViral DNA/RNA Kit (Qiagen, Hilden, Germany). PCR primer sequences were as follows: BKJCf: (5’-GCACTTTTGGGGGACCTAGT-3’), BKJCr: (5’-GGCAACATCCATTGAGGAG-3’), and JCV JCf: (5’-CCAAAGGGAGGGAACCTATATT-3’). PCR products were visualized on 2% agarose gels stained with ethidium bromide. When comparing samples by JCV status in subsequent analyses, JCV positivity was determined by PCR.

#### Microbiomes

Microbiomes were characterized from urine pellets containing bacteria (*i*.*e*., the fraction of the urine from which supernatants were removed as described above). Nucleic acids were extracted from pellet using the Qiagen Blood and Tissue Mini Kit (Qiagen, Hilden, Germany) per the manufacturer’s instructions. Polymerase chain reaction (PCR) of the 16s ribosomal RNA V4 region was then conducted following the Illumina^®^ 16S Metagenomic Library Preparation guidelines. The hypervariable (V4) region of the 16S rRNA SSU gene from each sample was amplified using primers for 515f: (5′-TCGTCGGCAGCGTCAGATGTGTATAAGAGACAGGTGYCAGCMGCCGCGGTAA-3′) and a revised 806Rb: (5′-GTCTCGTGGGCTCGGAGATGTGTATAAGAGACAGGGACTACNVGGGTWTCTAAT-3′) [34, 35]. Reactions contained 2 μL DNA template, 9.5 μL of Qiagen nuclease-free water, 12.5 μL of Qiagen HotStarTaq master mix, 0.5 μL of each 10 mM primer. Cycling conditions were as follows: initial denaturation step of 15 min at 95°C, followed by 35 cycles of 30s at 94°C, 30s at 60°C, 1 min at 72°C, and final extension of 10 min at 72°C. The library size was checked for a subset of samples using a Bioanalyzer High Sensitivity DNA Analysis chip (Agilent Technologies, Palo Alto, CA), followed by an indexing PCR using the Nextera XT Index Primer Kit (Illumina, Inc., San Diego, CA), following the manufacturer’s protocol. The size and quality of a representative subsample of the indexed libraries were examined using a Bioanalyzer High Sensitivity DNA Analysis chip and the PCR products were run on 2% agarose gels, extracting samples with Zymoclean gel DNA recovery kit (DNA Clean & Concentrator kit). Libraries were sequenced using a 600-cycle MiSeq Reagent Kit v3 (Illumina) for 2 × 300 on the Illumina MiSeq.

Raw sequence reads were processed using the R [36] package DADA2 [37]. To account for differing rates in the reduction of base quality in forward and reverse reads, reads were truncated at 240 and 200 base pairs long, respectively. Reads were then trimmed at the first appearance of a base with a quality score of less than 2 (if any were present). Reads with non-assigned bases (N) and that mapped to the PhiX sequencing standard were also removed at this stage. DADA 2 was then used to infer sequence variants (SVs) in the dataset using default parameters. Chimeric sequences were removed, and genus-level taxonomy was assigned by alignment to the SILVA ribosomal RNA database [38]. Following filtering and taxonomic assignment, our dataset comprised 2,336,970 reads originating from 3762 SVs.

Downstream processing and analysis was conducted in the R package phyloseq [39]. First, sequence variants belonging to the genera *Halomonas* and *Pseudoalteromonas* were removed because members of these genera are known contaminants of DNA extraction kits and laboratory reagents [40, 41]. Reads assigned as eukaryotic in origin were also removed at this stage. To avoid biases associated with extremely rare SVs, an abundance-based filter was applied. Sequence variants accounting for less than 100 total reads across all samples were removed. Removal of potential contaminants and ultra-rare SVs yielded a dataset of 2,258,897 reads from 1472 SVs. Prior to community analysis, read set sizes were normalized between samples by rarefaction to the smallest per-sample read set size (37,211), using the rarefy function in phyloseq. This resulted in a final dataset of 1,041,908 reads from 1472 SVs. Subsequent analysis of bacterial communities was performed with the aim of evaluating pairwise differences between two pairs of patients, those with and without LUTS and those with and without JCV (determined via PCR).

To examine differences in alpha diversity between groups, phyloseq was used to calculate each samples’ Shannon diversity index. Shannon indices were then converted into an effective number of species score (ENS) via exponentiation, as this is a more replicable and interpretable means of quantifying alpha diversity [42]. Wilcoxon sum ranked tests were performed in R to compare the ENS scores of the patient groups (*α* = 0.05). Differences in beta diversity, or bacterial community structure, were compared between the groups using the R package vegan [43] to produce a non-metric multi-dimensional scaling (NMDS) ordination using Bray-Curtis dissimilarity. Ordinations were performed across 2 dimensions (k = 2), which yielded a stress of fit value of 0.2. Differences in community structure between sample groups were tested statistically using permutational analysis of variance tests (PERMANOVA), implemented using the adonis function in vegan (*α* = 0.05). Each comparison was modeled separately and individual PEMANOVAs were constructed using 1000 permutations. To investigate which bacterial SVs differed most significantly in their abundance between patient groups, the linear discriminant analysis effect size method (LEfSe) was used [44]. LEfSe is a two-stage process that first determines which SVs are differentially enriched between comparison groups and then determines which of those differentially enriched SVs are consistently represented among individuals within a group. LEfSe was implemented using default parameters. Hierarchical clustering and a heatmap of all sequence variants based on the Bray-Curtis dissimilarity distance between samples were made with the R package heatmap2.

### Metabolomics

#### Urine sample preparation

Supernatants of urine samples were thawed on ice, vortexed to resuspend protein, centrifuged at 10,000 *g* for 10 min, and fractionated via 3 kDa centrifugal filters following the manufacturer’s protocol (Millipore, Burlington, MA). Briefly, urine samples (0.5 mL) were loaded into pre-washed filters and each flow-through metabolite fraction (< 3 kDa) was collected via centrifugation. The retained fraction (> 3 kDa) was washed twice with 0.45 mL ultrapure H_2_O (Fisher Scientific, Hampton, NH) and all flow-through fractions were combined and stored at -80°C. Osmolality was determined via freezing point depression (Advanced Instruments, Norwood, MA) and samples were normalized to this measure of solute concentration. Pre-acquisition normalization to osmolality performs well in global urine metabolomics analyses [45].

#### Ultrahigh performance liquid chromatography–tandem mass spectrometry

Global metabolomics with relative quantification was achieved via ultrahigh performance liquid chromatography coupled to tandem mass spectrometry (UHPLC-MS^2^). A Thermo Dionex UHPLC system coupled to a Thermo Q Exactive hybrid quadrupole-Orbitrap mass spectrometer was used for all analyses. Samples were ordered randomly, with blanks between each. Two method blanks were generated by collecting the flow-through fraction of ultrapure water to monitor sample preparation. A pooled quality control sample was injected every ten samples to monitor instrument stability. Samples were kept at 10°C in the autosampler until analysis. Analytes were separated in the reversed phase on a biphenyl UHPLC column (2.1 x 100 mm, 1.7 μm 100 Å) (Phenomenex, Torrance, CA). Column temperature was 30°C, flow rate was 0.3 mL/min, mobile phase A was H_2_O with 0.1% formic acid and mobile phase B was methanol with 0.1% formic acid. The UHPLC binary gradient was as follows: 0 min 5% B, 0–16.5 min 5–50% B (linear), 16.5–16.9 min 50–90% (linear), 16.9–18 min 90% B, 18–18.5 min 90–5% B (linear), 18.5–20 min 5% B. The mass spectrometer was operated in positive ion mode with stepped collision energy of 20, 30, and 40 NCE. Full scans from 100 to 1000 *m/z* were acquired with a resolution of 70,000, an automatic gain control target of 3E6, and a maximum injection time of 100 ms. Tandem MS scans were collected as follows: top 5 data-dependent acquisitions with 10 s dynamic exclusion at a resolution of 17,500 with an automatic gain control target of 2E5 and a maximum injection time of 50 ms.

#### Data processing and statistical analysis

Raw data files were processed using Thermo Compound Discoverer v3.0 software with ChemSpider and mzLogic compound databases. Retention times were aligned with a maximum shift of 2 min and compounds were grouped with a retention time tolerance of 0.2 min. Compounds were predicted with a maximum precursor mass of 5 kDa and mass tolerance of 5 ppm. Statistically significant changes were determined via volcano plot, using the Student’s *t*-test (α = 0.05) and a minimum fold-change of 50 percent. Significantly modulated mass-to-charge (*m/z*) features with assigned identities were manually categorized by role via the Human Metabolome Database (HMDB). Compounds were filtered to include those with known roles. Compounds representing likely exposures were excluded (*e*.*g*., drugs taken, dietary choices). When comparing samples by JCV status, JCV positivity was determined by PCR.

## Results and discussion

### Next-generation sequencing

#### Virome results

Analysis of 97,358,827 sequence reads (average 2,595,662 per sample) after quality trimming from 29 LUTS patients and 9 controls in the Wisconsin patient samples identified 14 of the 29 cases (48.3%) to be positive for the common neurotropic human polyomavirus JC virus (JCV; *Polyomaviridae*, *Betapolyomavirus*) (S1 Fig). No other viruses were identified in any sample. Based on metagenomics, the association between LUTS and JCV was statistically significant (odds ratio 17.8; Fisher’s exact one-tailed *p*-value = 0.0080).

Nested PCR testing for JCV of the same urine supernatants from Wisconsin was then conducted based on these results. PCR identified 15 of the 29 LUTS samples to be positive for JCV (51.7%) and also 3 of the 9 controls to be positive for JCV (33.3%). Based on PCR data, therefore, the association between LUTS and JCV became not statistically significant due to the classification by PCR of three control samples that had previously tested negative by metagenomics (odds ratio 2.1; Fisher’s exact two-tailed *p*-value = 0.45). To test whether urinary JCV prevalence was unique to Wisconsin, the same nested PCR was then applied to a sample group from Texas (*n* = 72). Twenty of 72 urine samples contained detectable JCV and no significant differences in prevalence were observed between the Wisconsin and Texas populations (S1 Table). Additionally, neither age nor body mass index were significantly associated with the presence of detectable JCV in urine (S1 Table).

JCV is the etiological agent of progressive multifocal leukoencephalopathy (PML), a deadly demyelinating disease most common in immunocompromised patients [46]. In general, polyomaviruses deregulate DNA repair enzymes, a critical defense against oxidative stress-related damage [47]. The JCV in particular causes DNA damage, and host responses to this damage may promote viral replication [48]. Interestingly, JCV may act synergistically with other agents, including environmental contaminants. For example, a mixture of the environmental contaminants polycyclic aromatic hydrocarbons increased oxidative DNA damage and the JCV prevented their repair [49]. JCV may play a role in neurodegenerative diseases via oxidative stress and dysregulation of glutathione [29].

Our results suggest a potential indirect role of JCV on LUTS. In the patient samples from Wisconsin, differences between results obtained from metagenomics and nested PCR likely reflect greater sensitivity of the nested PCR [33]. However, PCR targets only a small fragment of the viral genome, whereas metagenomics targets the entire viral genome and may thus be less prone to false positive results. Viruses often have indirect effects on clinical outcomes, and these may be highly contextual with significant “lag times” between initial viral infection and the appearance of clinical disease [50–52]. For example, some viruses can precipitate autoimmune responses that have clinical consequences years after initial viral infection [52, 53]. In the case of JCV, environmental factors can reactivate latent infection [54]. While JCV is best-understood in the context of PML, its role in other, less dramatic clinical diseases has not, to our knowledge, previously been explored. Given the virus’s ability to cause oxidative stress and inflammation-related processes [18, 19], it is plausible that JCV contributes to the onset or severity of LUTS. A limitation of our study is that we were able to detect only virus that was actively shed in urine. The site of replication of JCV in the urinary tract of LUTS patients and controls, plus any associated pathology or perturbations of cellular processes, would need to be determined prior to implicating JCV in the development of LUTS. Inter-tissue variability in JCV replication could explain some of the differences between our WI and TX samples. For example, it is unclear whether the urethra, which is an important microbial contributor to urine [8], supports JCV replication, but samples collected via catheter would be devoid of urethral inputs (*viz*. some TX cases and all TX controls). Given these limitations, validation-phase studies should be conducted to assess the tissue tropism of JCV along the urinary tract.

#### Microbiome results

**Fig 2:**
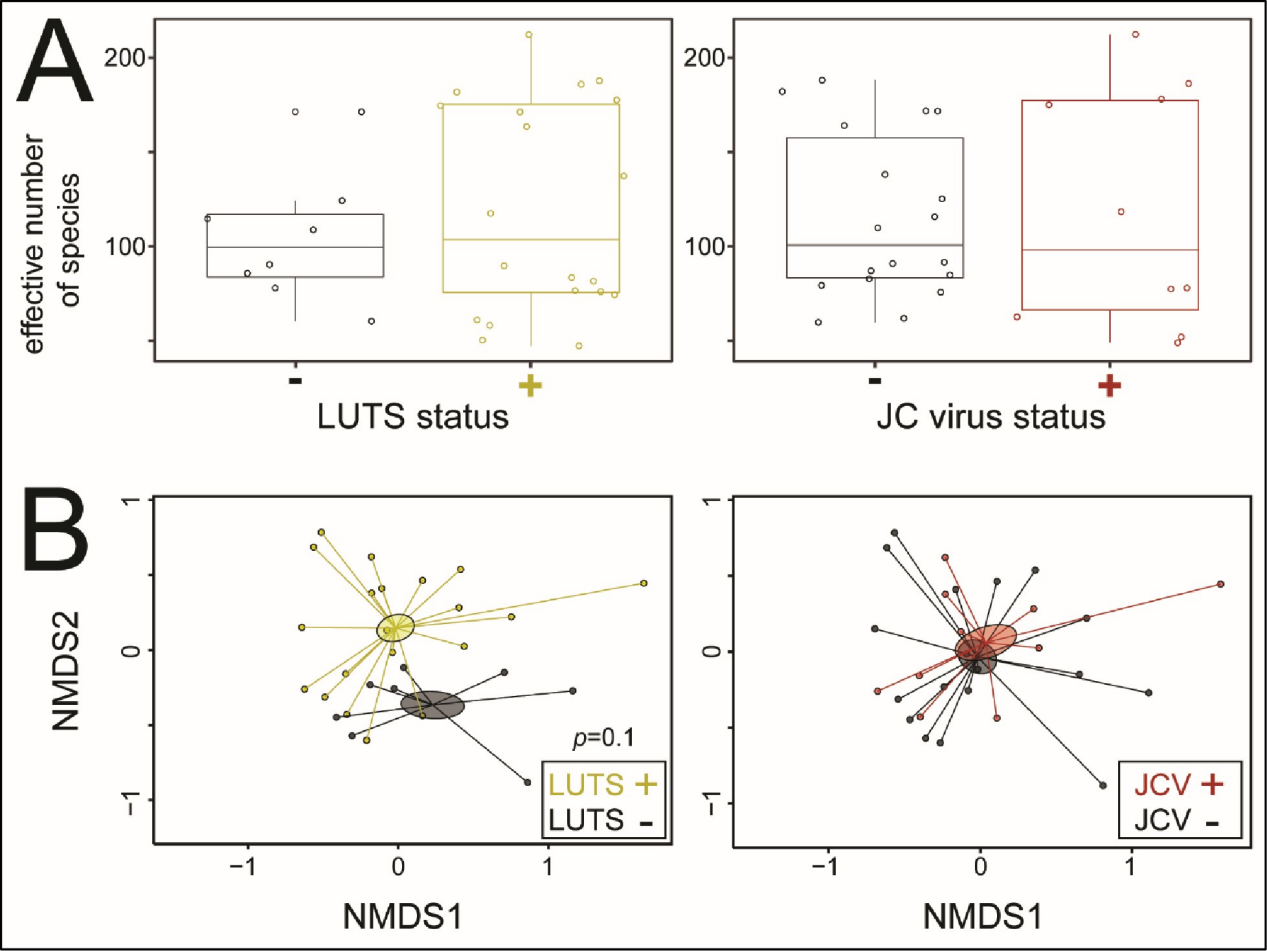
Urine microbiomes: comparisons of bacterial communities observed in LUTS+ *vs* LUTS- samples and JCV+ *vs* JCV- samples. **A**: alpha diversity–the number of species present regardless of community structure or taxonomy. The effective number of species (ENS; *y*-axis) of each sample (points) is displayed over a box and whisker plot showing the distribution of the ENS values for each comparison group. Statistical analysis showed no significant differences in either LUTS+/- or JCV+/- comparisons (Wilcoxon test, *p* = 0.78 and *p* = 0.87, respectively), and **B**: beta diversity–differences in community structure between LUTS+/- and JCV+/- comparison groups. Plots show a non-metric multi-dimensional scaling based on Bray-Curtis dissimilarity distances between samples: statistical comparison of group centroids using permutational analysis of variance (PERMANOVA) showed no significant differences in LUTS+/- or JCV+/- comparison groups (PERMANOVA, *p* = 0.11 and *p* = 0.67, respectively).

**Fig 3:**
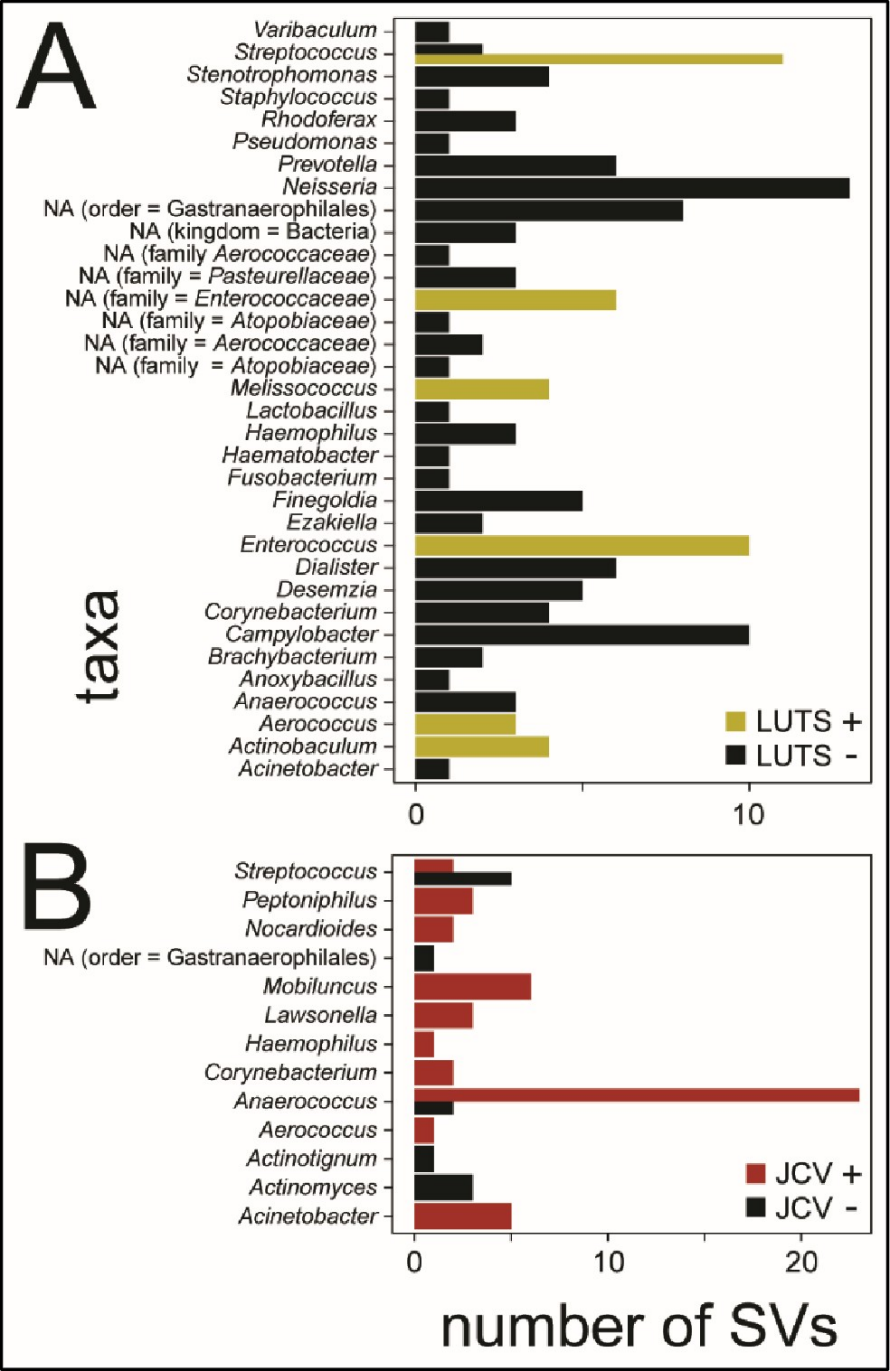
Urine microbiomes: Unique bacterial genera found to be biomarkers of either member of a comparison group are shown on the *y*-axis and the number of sequence variants belonging to each genera on the *x*-axis; summary of results from linear discriminant effect size analysis (LEfSe). **A**: LUTS positive and negative, and **B**: JC virus positive and negative. Note increased *Streptococcus* and *Enterococcus* sequence variants in LUTS+ samples and increased *Anaerococcus* sequence variants in JCV+ samples.

To assess the possibility of bacterial microbiome dysbiosis related to LUTS and JCV infection, we compared bacterial microbiomes of LUTS *vs* control and JCV positive *vs* negative samples. Alpha diversity, a measure of the number of bacterial species present regardless of community structure or taxonomy showed no significant differences in either LUTS *vs* control (Wilcoxon test *p* = 0.78) or JCV positive *vs* negative (Wilcoxon test *p* = 0.87) (Fig 2). Beta diversity, a measure of differences in bacterial community structure, also showed no overall differences between LUTS *vs* control (PERMANOVA *p* = 0.11) or JCV positive *vs* negative (PERMANOVA *p* = 0.67) (Fig 2). Linear discriminant analysis Effect Size (LEfSe) measures the divergence in abundance between sample groups and then examines the within-group consistency in abundance to find which bacterial sequence variants are biomarkers of a particular group. In LUTS *vs* control samples, LEfSe analysis showed a number of bacterial taxa with increased representation in LUTS *vs* control samples (S2 Fig). For example, *Streptococcus* and *Enterococcus spp*. were relatively increased in LUTS samples, while *Neisseria* and *Campylobacter spp*. were relatively increased in controls (Fig 3). Similarly, a number of species were increased in both JCV positive and negative samples (S3 Fig). For example, *Anaerococcus spp*. were highly increased in the JCV positive samples (Fig 3). Hierarchical clustering with heatmap analysis showed overall homogeneity with respect to LUTS status and JCV status, perhaps due to the taxonomic resolution of the identified SVs (S4 Fig). Finally, 10 of 39 total urine samples contained no detectable 16S rDNA and were thus not carried forward.

Dysbiosis in male LUTS is poorly understood. A previous study found no association between bacterial community composition and LUTS symptom severity [15]. Here, we find similar results for the overall diversity and composition of bacterial communities. However, when considering differences in the abundance of individual bacterial taxa between groups using LEfSe, we were able to detect overabundances of certain taxa between patient groups. In particular, *Streptococcus* and *Enterococcus spp*. were increased in LUTS-positive samples. Both of these taxa are common in urinary tract infection [55, 56], raising the possibility that they may also play a role in LUTS, either as a cause or consequence. With regard to JCV associations, *Anaerococcus spp*. were substantially increased in JCV positive samples. While little is known about a potential connection between *Anaerococcus spp*. and JCV, these bacteria associate with prostate cancer, ascribed to their possible pro-inflammatory role in the prostate [57]. These results again suggest a possible polymicrobial etiology for LUTS (at least in some cases) that may operate through inflammation and immunity. It is important to note that the microbial signals detected in these analyses likely arose from tissues relevant to LUTS (*e*.*g*., urethra, prostate, possibly bladder or kidney), but that the specific tissues of origin remain unknown.

### Metabolomics of JCV

**Fig 4:**
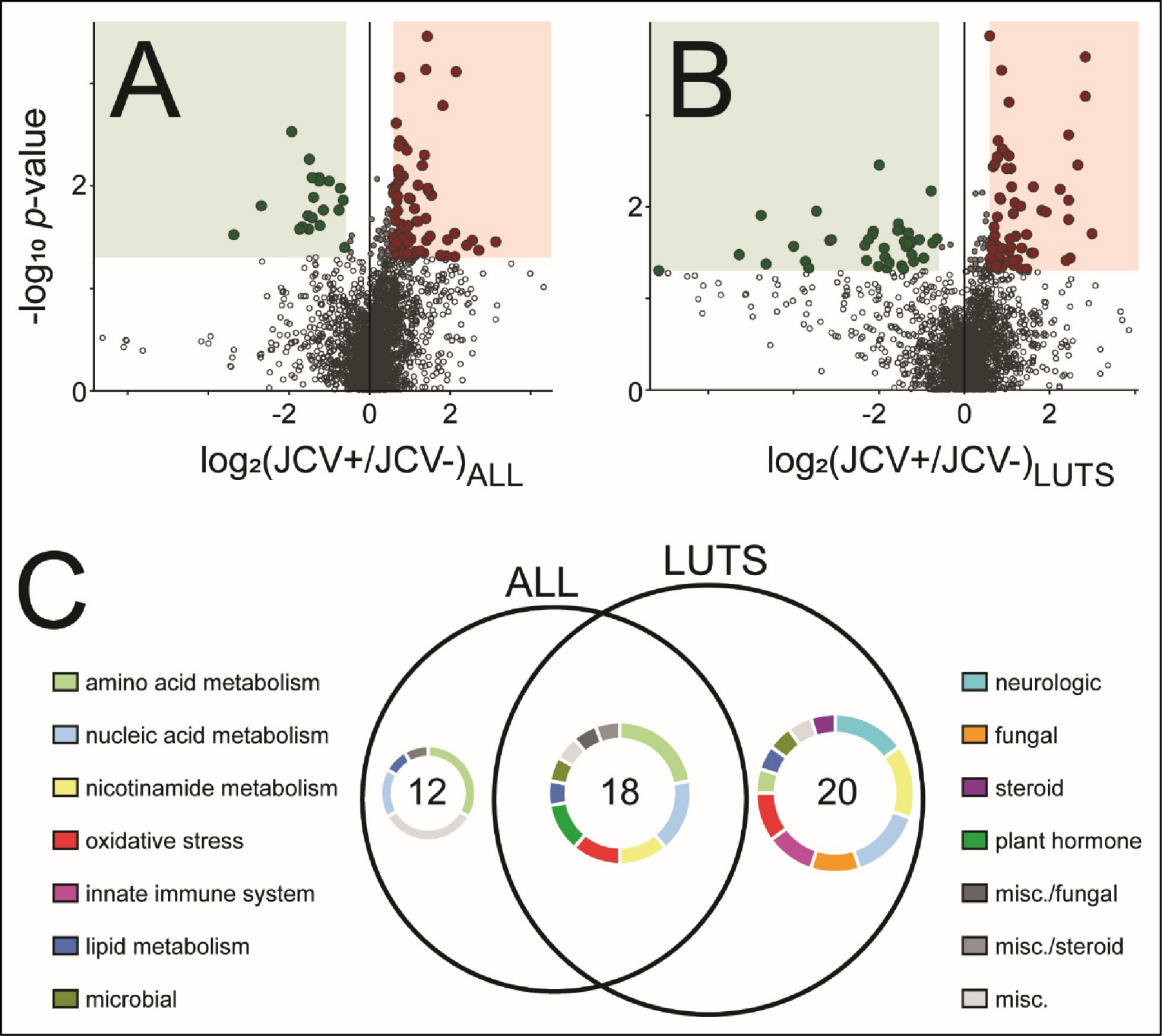
Urine metabolomes of JC virus in **A:** all samples and **B:** LUTS samples only. Volcano plots show 3079 and 2658 total *m/z* features, respectively; enlarged dots indicate significantly modulated features (Student’s *t*-test *p*-value < 0.05 and fold-change ≥ 50%); red indicates increase with JC virus, green indicates decrease with JC virus. **C:** Venn diagram of significantly modulated features shows the urine metabolome of JC virus in LUTS patients is larger and represents more roles, including neurologic, fungal, oxidative stress, and innate immune system (filtered by known compounds with known roles).

Urine metabolomics of JCV in all samples and in LUTS samples identified 3079 and 2658 *m/z* features, respectively (Fig 4). After filtering for known compounds with known roles and excluding hits related to behavioral factors, a total of 30 compounds in all samples and 38 compounds in LUTS samples were significantly different when comparing JCV positive and negative samples (Fig 4). Manual annotation via HMDB showed JCV positivity in all samples had more compounds related to amino acid metabolism and other miscellaneous processes than did LUTS only samples (Fig 4). The urine metabolome of JCV in LUTS patients had more compounds related to LUTS-relevant processes, including oxidative stress, neurologic function, and the innate immune system than did all samples (Fig 4).

Prior work has established associations between oxidative stress and both LUTS [58] and viral infection [19]. Here, oxidative stress-related compounds, *L*-glutathione (oxidized) and carnosine, were significantly increased in samples with JCV from LUTS patients as well as all samples (Table 2, S1 Table). Additional oxidative stress-related molecules, *L*-pyroglutamic acid and *N*-acetyl-*L*-carnosine, were significantly dysregulated only in the LUTS samples (Table 2). Glutathione plays a critical role in the inactivation of many reactive species in its reduced form [59] and the oxidized form was found increased in the urine of JCV positive samples regardless of LUTS status, suggesting increased oxidative stress in these samples. Carnosine is a radical scavenger and metal chelator [60] and was also significantly dysregulated (increased) with JCV positivity in both all samples and LUTS samples. *N*-acetyl-*L*-carnosine is a more potent antioxidant than carnosine [61], and was increased with JCV positivity only in LUTS samples. *L*-pyroglutamic acid accumulates under conditions of glutathione synthetase deficiency [62]. Together with increased oxidized glutathione, the increase in *L*-pyroglutamic acid suggests JCV positivity in LUTS is associated with an oxidative stress imbalance beyond the capacity of endogenous antioxidant mechanisms, potentially contributing to the symptoms. Given this corroborating evidence, these compounds may be useful as biomarkers for patient stratification, but care should be taken regarding over-interpretation of these discovery-phase data. Validation-phase, targeted analyses in new and larger sample groups should first be performed and would appear warranted.

**Table 2:** Significantly modulated urine metabolites associated with JC virus when comparing LUTS samples only; Student’s *t*-test *p*-value < 0.05 and fold-change ≥ 50%. Metabolite roles were manually annotated using the Human Metabolome Database.

category	m/z feature identity	formula	molecular weight	retention time (min)	fold-change log_2_ (JCV+/JCV-)	BH *p*-value
nucleic acid metabolism	xanthine	C5 H4 N4 O2	152.0334	1.258	0.83	4.65E-02
	5-methylcytosine	C5 H7 N3 O	125.0590	1.302	0.81	1.64E-02
	uracil	C4 H4 N2 O2	112.0276	1.113	0.67	4.73E-02
	7-methylguanine	C6 H7 N5 O	165.0651	3.179	0.66	4.16E-02
	5-methylcytidine	C10 H15 N3 O5	257.1011	1.312	0.62	4.65E-02
	1-methylhypoxanthine	C6 H6 N4 O	150.0542	3.858	-1.98	1.64E-02
amino acid metabolism	*N*-phenylacetylglutamic acid	C13 H15 N O5	265.0950	10.35	1.26	4.66E-02
	*N*-(indol-3-ylacetyl)glutamine	C15 H17 N3 O4	303.1219	9.467	1.05	1.40E-02
	asn-pro	C9 H15 N3 O4	229.1062	1.436	-0.73	4.15E-02
	hydantoin-5-propionic acid	C6 H8 N2 O4	172.0484	1.475	-0.77	2.16E-02
	xanthurenic acid	C10 H7 N O4	205.0352	4.464	-1.31	4.15E-02
nicotinamide metabolism	nicotinamide 1-oxide	C6 H6 N2 O2	138.0429	1.373	1.63	2.11E-02
	6-methylnicotinamide	C7 H8 N2 O	136.0636	0.948	1.15	3.06E-02
	arecoline	C8 H13 N O2	155.0947	1.21	0.71	3.95E-02
	nicotinamide	C6 H6 N2 O	122.0483	1.399	0.62	4.73E-02
	5-aminonicotinic acid	C6 H6 N2 O2	138.0429	4.144	-1.87	4.15E-02
oxidative stress	*L*-pyroglutamic acid	C5 H7 N O3	129.0428	2.975	2.67	1.64E-02
	*L*-glutathione oxidized [Δmass: -34.0290 Da]	C26 H32 N8 O8 P2	646.1810	2.369	1.09	4.11E-02
	carnosine [Δmass: -47.0046 Da]	C14 H15 N3 O3	273.1112	5.051	0.77	4.15E-02
	*N*-acetyl-*L*-carnosine	C11 H16 N4 O4	268.1168	1.079	0.68	1.64E-02
neurologic	*N*-acetyl-1-aspartylglutamic acid [Δmass: 85.0342 Da]	C8 H13 N O4 S	219.0565	2.974	1.83	3.06E-02
	5-hydroxyindole-3-acetic acid	C10 H9 N O3	191.0583	11.104	1.59	4.38E-02
	*N*-acetylhistamine	C7 H11 N3 O	153.0901	1.083	0.64	4.73E-02
fungal	myriocin	C21 H39 N O6	401.2777	17.882	-1.46	4.73E-02
	(2*Z*)-8-hydroxy-2-octene-4,6-diynoic acid	C8 H6 O3	150.0317	5.243	-1.50	4.03E-02
	1-[2,4-dihydroxy-3-(2-hydroxyethyl)-6-methoxyphenyl]-1-butanone	C13 H18 O5	254.1155	17.796	2.99	4.05E-02
microbial	3-methylene-3H-indole	C9 H7 N	129.0579	9.469	0.89	1.24E-02
	4-pyridoxic acid [Δmass: -158.0216 Da]	C14 H15 N O9	341.0747	3.898	0.88	4.73E-02
innate immune	formyl-*L*-methionine	C6 H11 N O3 S	177.0459	1.127	1.92	3.06E-02
	succinate	C4 H5 O4	117.0193	1.365	-4.64	4.73E-02
plant hormone	indole-3-acetic acid	C10 H9 N O2	175.0633	10.338	1.09	1.64E-02
	indole-3-acetic-acid-*O*-glucuronide	C16 H17 N O8	351.0953	10.333	0.97	1.64E-02
lipid metabolism	*O*-octanoyl-*L*-carnitine	C15 H29 N O4	287.2095	15.19	1.12	2.11E-02
	9-decenoylcarnitine	C17 H31 N O4	313.2253	17.869	0.72	4.05E-02
steroid	boldenone [Δmass: 55.1368 Da]	C9 H13 N O4 S	231.0565	2.498	-3.99	4.15E-02
	1,4-androstadiene-3,17-dione	C19 H24 O2	284.1776	17.089	-7.15	4.99E-02
misc	pyridoxal	C8 H9 N O3	167.0583	1.414	1.21	4.05E-02
	5-indolol	C8 H7 N O	133.0528	7.38	1.00	1.64E-02

Neurologic dysfunction is an important component of LUTS pathogenesis [63] and is a critical component of PML caused by JCV [46]. Compounds related to neurologic function were significantly dysregulated (increased) with JCV positivity in the LUTS samples, including *N*-acetyl-1-aspartylglutamic acid, 5-hydroxyindole-3-acetic acid, and *N*-acetylhistamine (Table 2). *N*-acetyl-1-aspartylglutamic acid is among the most abundant neuropeptides and its dysregulation associates with neurodegenerative diseases [64]. *N*-acetylhistamine is a metabolite of histamine, a molecule with roles in neurologic function and inflammation, among other processes [65]. 5-hydroxyindole-3-acetic acid is a metabolite of serotonin, which is increased in response to inflammation [66]. These findings are consistent with the idea that JCV, a neurotropic virus known for its rare but severe effects on the nervous system [46], may affect urinary tract diseases such as LUTS through neurologic pathogenic pathways.

Inflammation is another likely contributor to LUTS. Compounds related to the innate immune system that were significantly dysregulated (increased) with JCV positivity in LUTS samples include formyl-*L*-methionine and succinate. Interestingly, formyl-modified peptides are pro-inflammatory molecules of bacterial or host mitochondrial origin and signal in the pathogen-associated molecular pattern pathway of the innate immune system via formyl peptide receptors [67]. Finally, succinate is a molecule with many roles, including oxidative stress and inflammation [68].

## Conclusion

The etiology of LUTS is likely complex and multifactorial, varying from patient to patient. However, growing evidence suggests that microbes may play a role. Viruses in particular are capable of perturbing important processes in other diseases, but their role in LUTS has, to our knowledge, not previously been explored. We found the neurotropic human virus, JCV, to be variably associated with LUTS, with the overabundance of certain bacterial taxa, and with increased abundance of metabolomic markers of oxidative stress and inflammation. The precise contribution of JCV to LUTS development and severity—if any—remains uncertain, but changes in the urinary microbiome and metabolome associated with JCV merit further investigation. The current study is largely hypothesis-generating in nature and would benefit from future investigation of the urinary tropism and cellular pathology of JCV in humans or in a suitable animal model. Additionally, prospective, validation-phase studies quantitatively targeting JCV and markers of oxidative stress in urine samples or benign prostate biopsy samples could help shed light on the potential role of JCV in LUTS. Given the multifactorial nature of LUTS, future efforts to identify potential etiological agents would benefit from stratifying patients into sub-groups based on clinical observations (*e*.*g*., prostate volume, urodynamics).

## Supporting information

S1 Fig Next-generation sequencing: JC virus sequence coverage shows full genome coverage. (PDF)

S2 Fig LEfSe plot: LUTS positive versus LUTS negative. (PDF)

S3 Fig LEfSe plot: JC virus positive versus JC virus negative. (PDF)

S4 Fig Hierarchical clustering and heatmap of all sequence variants. (PDF)

S1 Table Association of JC virus with age or body mass index within-group and comparison of prevalence between the WI and TX sample groups. The presence of urinary JCV was not significantly associated with age or body mass index (BMI) within either sample group, and the prevalence of JCV was not significantly different between the regionally distinct sample groups.
(PDF)

S2 Table Significantly modulated urine metabolites associated with JC virus when comparing all samples. Significantly modulated urine metabolites associated with JC virus when comparing all samples; Student’s *t*-test *p*-value < 0.05 and fold-change ≥ 50%. Metabolite roles manually annotated via Human Metabolome Database.
(PDF)

S3 Table Metabolomics results, full list: ALL samples. (XLSX)

S4 Table Metabolomics results, full list: LUTS only samples. (XLSX)
