# Supplementary figures and images for: A multi-omic investigation of male lower urinary tract symptoms: Potential role for JC virus
Source: PLoS One. 2021 Feb 25;16(2):e0246266. doi: 10.1371/journal.pone.0246266 (PMC7906371; doi:10.1371/journal.pone.0246266)

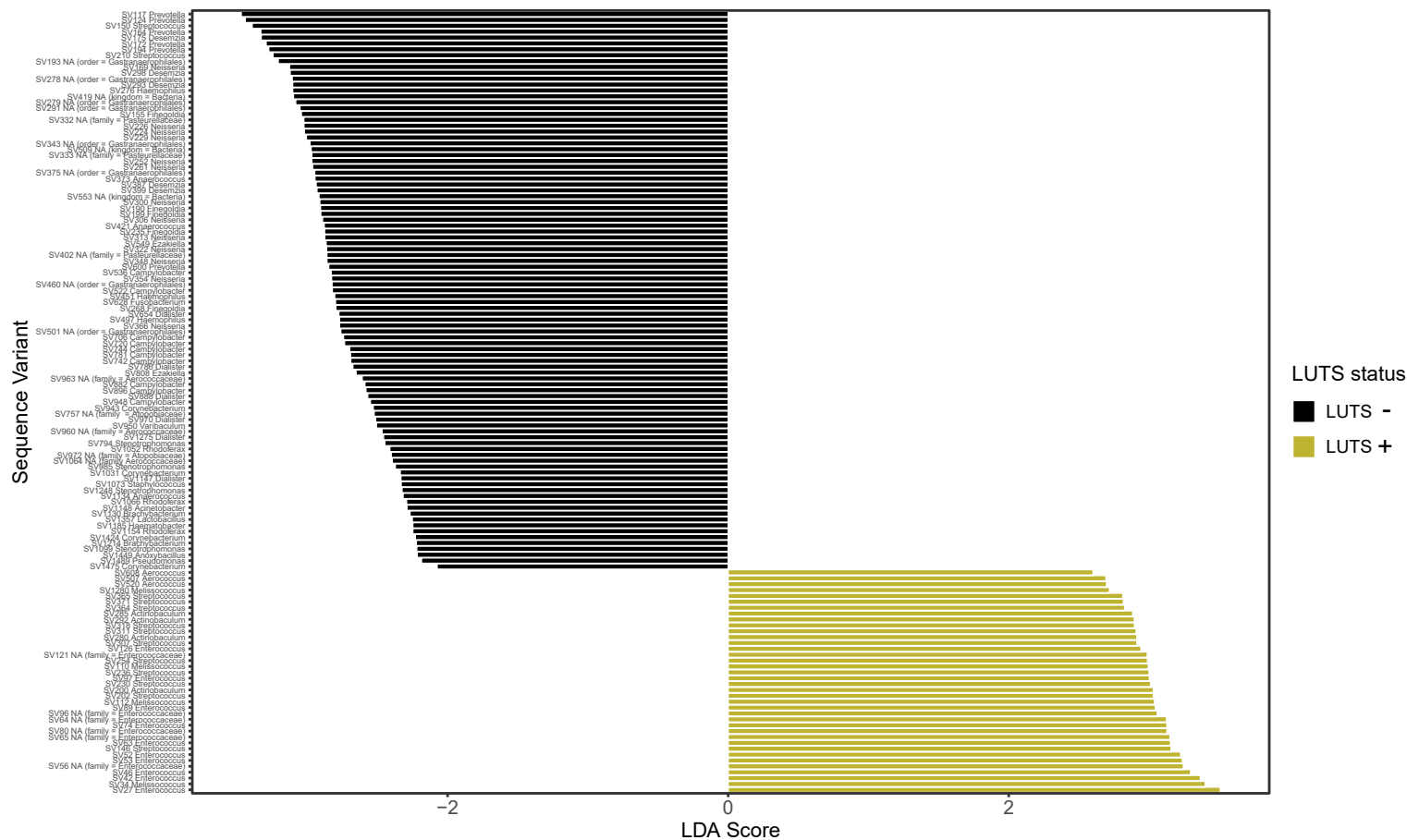

Figure S-2. LEfSe plot: LUTS positive versus LUTS negative

Supplement: S2 Fig — (PDF) [file pone.0246266.s002.pdf]

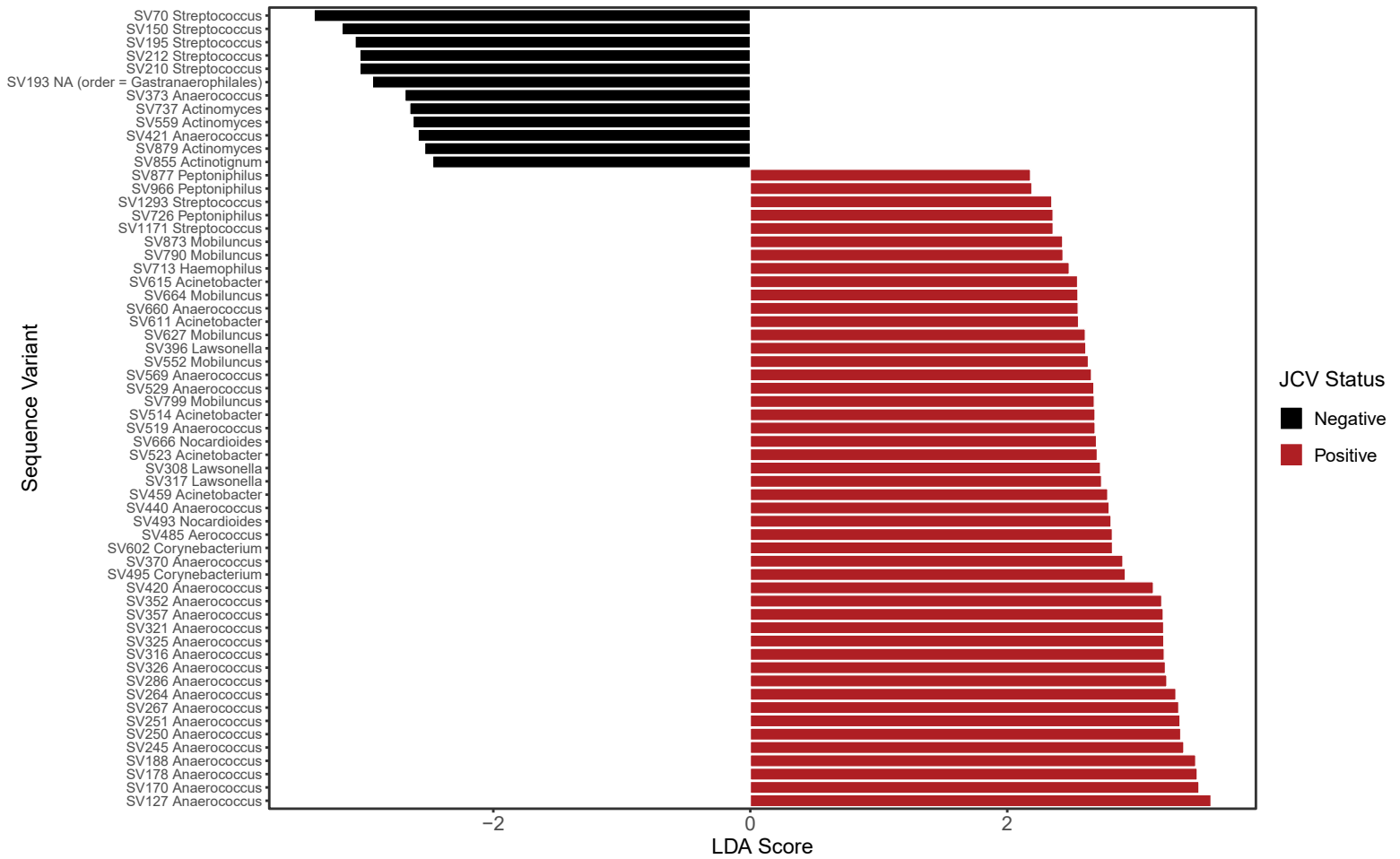

Supplement: S3 Fig — (PDF) [file pone.0246266.s003.pdf]
