# Supplementary material for: A multi-omic investigation of male lower urinary tract symptoms: Potential role for JC virus
Source: PLoS One. 2021 Feb 25;16(2):e0246266. doi: 10.1371/journal.pone.0246266 (PMC7906371; doi:10.1371/journal.pone.0246266)
Supplement: S4 Fig — (PDF) [file pone.0246266.s004.pdf]

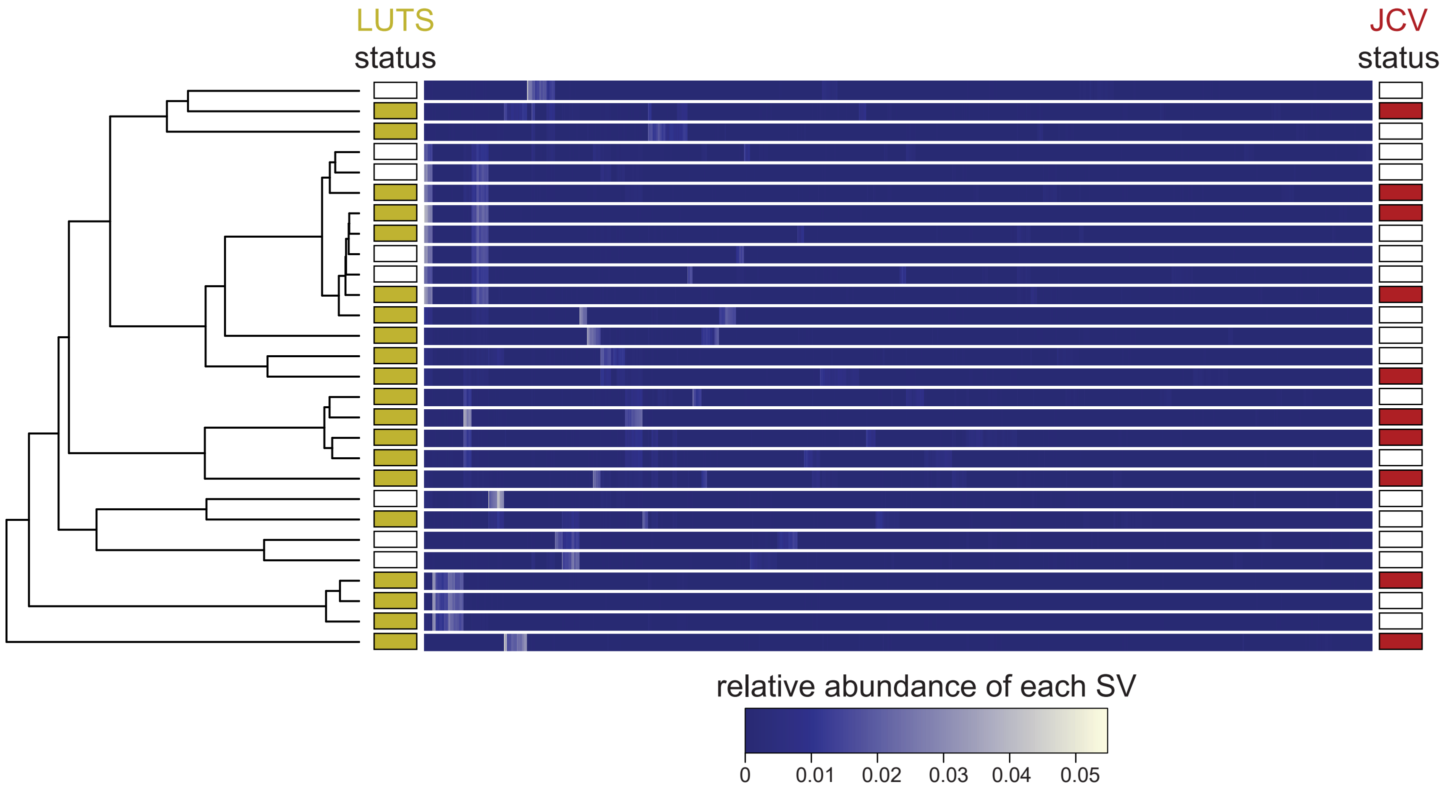

Figure S-4. Hierarchical clustering and heatmap of all sequence variants (SV; 1400 total) for all samples with detectable 16 rDNA, with both LUTS status and JCV status indicated.
