# Supplementary material for: A multi-omic investigation of male lower urinary tract symptoms: Potential role for JC virus
Source: PLoS One. 2021 Feb 25;16(2):e0246266. doi: 10.1371/journal.pone.0246266 (PMC7906371; doi:10.1371/journal.pone.0246266)
Supplement: S1 Table — The presence of urinary JCV was not significantly associated with age or body mass index (BMI) within either sample group, and the prevalence of JCV was not significantly different between the regionally distinct sample groups. (PDF) [file pone.0246266.s005.pdf]

Table S-1. The presence of urinary JCV was not significantly associated with age or body mass index (BMI) within either sample group[a], and the prevalence of JCV was not significantly different between the regionally distinct sample groups[b].

sample group	variable[a]	odds ratio (95% confidence)[a]	*p*-value[a]	$n_{JCV\text{-}positive}$ / n_{total}	*p*-value[b]
Wisconsin	age	1.01 (0.97-1.05)	0.67	18/38	0.057
	BMI	1.20 (0.99-1.44)	0.06		
Texas	age	1.01 (0.98-1.04)	0.51	20/72	
	BMI	1.03 (0.92-1.14)	0.64		

[a] Binomial logistic regression analysis of association between age or BMI with presence of JCV.
[b] Fisher's exact two-tailed test comparing the proportion of JCV-positive samples in each group.
