# Supplementary material for: A multi-omic investigation of male lower urinary tract symptoms: Potential role for JC virus
Source: PLoS One. 2021 Feb 25;16(2):e0246266. doi: 10.1371/journal.pone.0246266 (PMC7906371; doi:10.1371/journal.pone.0246266)
Supplement: S2 Table — Significantly modulated urine metabolites associated with JC virus when comparing all samples; Student’s t-test p-value < 0.05 and fold-change ≥ 50%. Metabolite roles manually annotated via Human Metabolome Database. (PDF) [file pone.0246266.s006.pdf]

Table S-2. Significantly modulated urine metabolites associated with JC virus when comparing all samples; Student's *t*-test *p*-value < 0.05 and fold-change ≥ 50%. Metabolite roles manually annotated via Human Metabolome Database.

category	m/z feature identity	formula	molecular weight	retention time (min)	fold-change $\log_2$ (JCV+/JCV-)	*p*-value
amino acid metabolism	val-leu	C11 H22 N2 O3	230.1630	1.582	1.91	4.86E-02
	N-phenylacetylglutamic acid	C13 H15 N O5	265.0950	10.35	1.15	4.38E-02
	N-(indol-3-ylacetyl)glutamine	C15 H17 N3 O4	303.1219	9.465	0.83	4.06E-03
	N-phenylacetylglutamine [Δmass: -6.0468 Da]	C13 H22 N2 O4	270.1578	15.357	0.74	2.91E-02
	gamma-glutamylleucine	C11 H20 N2 O5	260.1372	9.776	0.69	1.76E-02
	asparaginyl-4-hydroxyproline	C9 H15 N3 O5	245.1010	0.88	0.68	4.64E-02
	hydantoin-5-propionic acid	C6 H8 N2 O4	172.0484	1.472	-0.75	1.74E-02
	xanthurenic acid	C10 H7 N O4	205.0352	4.461	-1.23	9.00E-03
nucleic acid metabolism	hypoxanthine	C5 H4 N4 O	136.0386	1.488	1.12	4.75E-02
	*N*2-methylguanosine	C11 H15 N5 O5	297.1073	3.181	0.78	2.36E-02
	xanthine	C5 H4 N4 O2	152.0334	1.255	0.64	1.01E-02
	7-methylguanine	C6 H7 N5 O	165.0651	3.178	0.61	1.89E-02
	1-methylhypoxanthine	C6 H6 N4 O	150.0541	3.858	-1.92	2.99E-03
oxidative stress	carnosine [Δmass: -47.0046 Da]	C14 H15 N3 O3	273.1112	5.051	0.96	2.44E-02
	L-glutathione oxidized [Δmass: -34.0290 Da]	C26 H32 N8 O8 P2	646.1810	2.366	0.94	3.13E-02
microbial	3-methylene-3H-indole	C9 H7 N	129.0579	9.468	0.76	8.68E-04
	3,4-dihydroxystyrene	C8 H8 O2	136.0525	5.18	-1.67	2.54E-02
plant hormone	indole-3-acetic acid	C10 H9 N O2	175.0633	10.338	0.98	1.31E-02
	indole-3-acetic-acid-O-glucuronide	C16 H17 N O8	351.0954	10.333	0.83	3.93E-03
nicotinamide metabolism	nicotinamide 1-oxide	C6 H6 N2 O2	138.0429	1.37	1.21	9.99E-03
	6-methylnicotinamide	C7 H8 N2 O	136.0636	0.945	1.02	1.34E-02
lipid metabolism	*O*-octanoyl-*L*-carnitine	C15 H29 N O4	287.2095	15.189	1.41	7.37E-04
	(4*S*)-4-[(2*E*)-2-octenoyloxy]-4-(trimethylammonio)butanoate	C15 H27 N O4	285.1939	14.606	0.99	3.30E-02
misc	1-[2,4-dihydroxy-3-(2-hydroxyethyl)-6-methoxyphenyl]-1-butanone	C13 H18 O5	254.1155	17.79	1.77	4.87E-02
	5-indolol	C8 H7 N O	133.0528	7.379	1.44	3.47E-04
	N-acetylneuraminic acid [Δmass: -1.0203 Da]	C12 H22 O9	310.1263	10.254	1.12	4.62E-02
	4-hydroxyphenylacetylglycine	C10 H11 N O4	209.0688	11.16	0.93	3.60E-02
	creatinine [Δmass: -136.0383 Da]	C8 H15 N3 O6	249.0972	1.173	0.84	4.57E-02
	tiglylcarnitine	C12 H21 N O4	243.1470	7.04	0.63	3.73E-02
	boldenone [Δmass: 55.1368 Da]	C9 H13 N O4 S	231.0565	2.497	-3.35	3.03E-02
