# Supplementary material for: A multi-omic investigation of male lower urinary tract symptoms: Potential role for JC virus
Source: PLoS One. 2021 Feb 25;16(2):e0246266. doi: 10.1371/journal.pone.0246266 (PMC7906371; doi:10.1371/journal.pone.0246266)
Supplement: S1 Fig — (PDF) [file pone.0246266.s001.pdf]

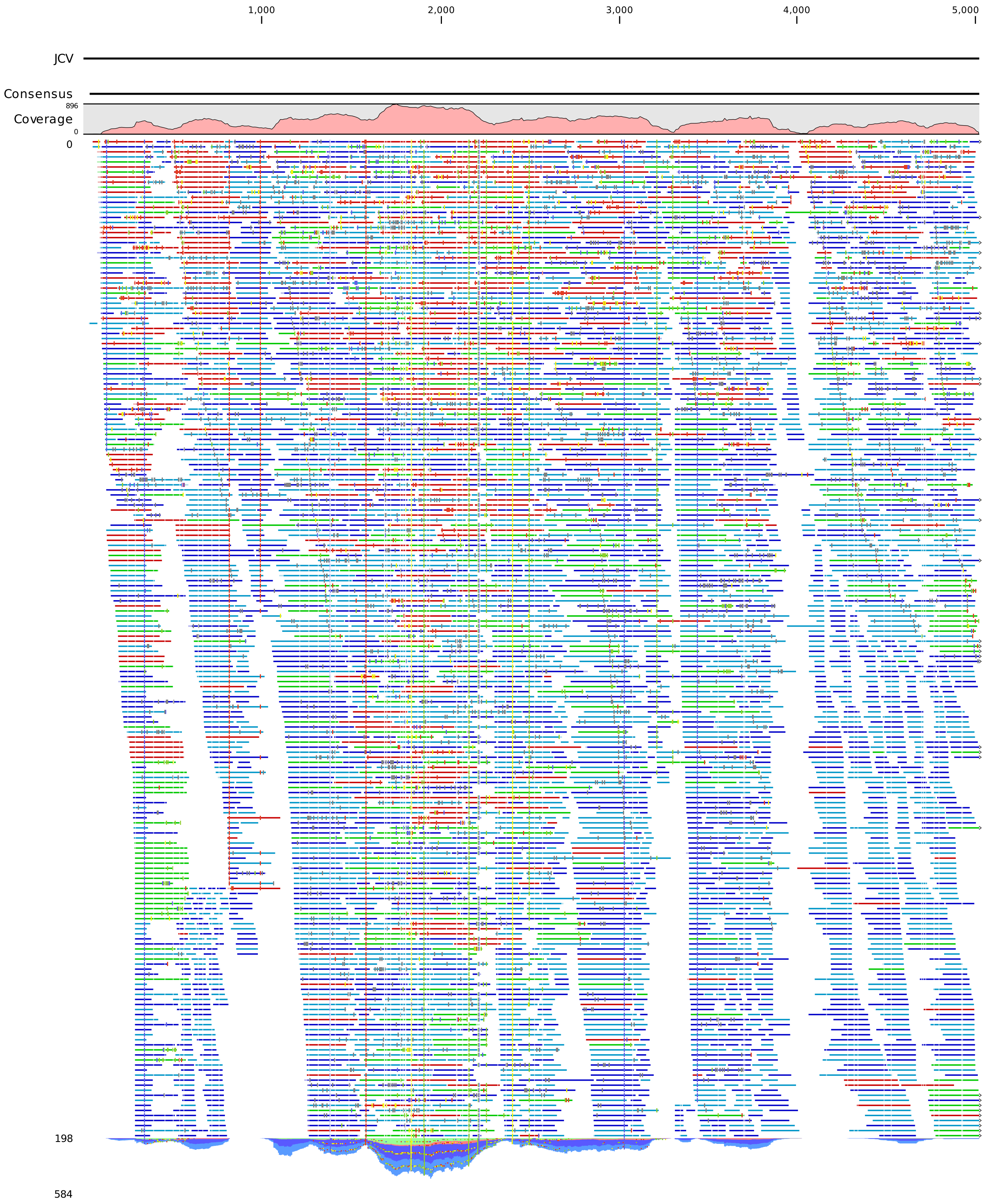

Figure S-1. Next-generation sequencing: JC virus sequence coverage shows full genome coverage
